# Definition of Tinnitus

**DOI:** 10.3390/audiolres12030029

**Published:** 2022-05-23

**Authors:** Aldo Messina, Alessandro Corvaia, Chiara Marino

**Affiliations:** 1 Department of Biomedical, Dental, Morphological and Functional Imaging Sciences, University of Messina, 98100 Messina, Italy; 2 Regina Margherita Otoneurological Center, 90145 Palermo, Italy; alexander323@hotmail.it (A.C.); chiara.marino1992@gmail.com (C.M.)

**Keywords:** tinnitus, sensation, perception, dysperception, hallucination, hallucinosis, pseudo-hallucination, delusional behaviors, inflammation, neuroplasticity, neuroplasticity dysfunction, gate keeper system, somatosound, gestalt

## Abstract

Tinnitus is generally defined as the perception of sound in the absence of vibration of an external elastic body. If this definition appears useful to differentiate tinnitus from somatosounds, it is not suitable for distinguishing it from psychiatric hallucinations. Nor does this solution define a temporal limit of duration of the perception, which is important for distinguishing pathological tinnitus from those occasional noises that we all perceive from time to time. A complete definition appears necessary not only to achieve homogeneity in epidemiological studies but also to set up correct and personalized therapeutic schemes. An analogy with neuropsychiatric studies and, in particular, the concept of auditory hallucinosis are proposed by the authors to define tinnitus. According to the authors, tinnitus is auditory hallucinosis, and similarly, vertigo is spatial hallucinosis.

## 1. Introduction 

Subjective Tinnitus is defined as the perception of a sound in the absence of any external vibratory stimulation. Now, everyone accepts the fact that this symptom mainly derives from activity within the Central Nervous System. Though tinnitus originates from a peripheral hearing impairment, it involves the central nervous system.

This “phantom sound” is mostly described as “ringing in the ears”.

P.J. Jastreboff describes it as the “perception of a sound in the absence of external sound stimulation” [1,2].

Patients understand that tinnitus is a “disorganized acoustic perception, not actually produced by any sound source, neither inside nor outside our body”.

These descriptions of tinnitus do not appear exhaustive, at least because they do not distinguish between tinnitus and psychiatric hallucinations. The latter can also be classified as “sound in the absence of stimulation”, and, as we will see later, the distinctive character cannot be represented by the organization of the perception. A person with tinnitus can also report quite complex perceptions, including musical ones and, therefore, well-structured ones, without being a psychiatric patient. We will examine this issue further.

We need a more exhaustive definition of tinnitus for correctly identifying the clinical picture, to avoid confusion that is also reflected in both epidemiological and therapeutic statistical studies and finally to indicate new therapeutic paths.

## 2. Materials and Methods

We wish to propose a definition of tinnitus that on the one hand can be exhaustive in relation to the current knowledge of the pathology and, on the other, is suitable for defining a nosological framework in the context of other similar pathologies of the Central Nervous System.

Hence, it should not seem superfluous to make some general neurological premises.

Sensation is a sensory experience that involves the activation of one of the many sense organs we are endowed with. It describes bottom-up a type of reality that is in any case bound to the working of our receptors. If an object vibrates at a frequency of 25,000 Hz, since man’s auditory sensory organ does not decode this frequency, our sensation will be of a silent “reality”. Conversely, for other living beings, such as dogs, whose cochlear receptor also transduces this frequency, the same stimulation will turn into a reality consisting in sound. Sensation is therefore an exclusively bottom-up experience, mediated by the sensory receptor.

When the sensory flow reaches the corresponding cerebral cortical areas and we become aware of it, the perceptual phenomenon is activated. This is a psychic process and therefore a subjective one, since the cortical areas operate a cross-modal synthesis of sensory data, giving meaning to forms. Today it is believed that the perceptive act is the consequence of a synthesis operated not by a single cortical area but by the organization of multiple sensory data in a complex experience involving the whole organism [3]. 

These principles are the basis of the Gestalt theory, a term that derives from the German *Gestaltpsychologie*, ‘psychology of form’ or ‘representation’, a psychological current focused on the themes of perception and experience [4]. 

At this point, it seems legitimate to wonder whether there may be a perceptive act (tinnitus), in the absence of sensory activation (“phantom sound”). However, it is undeniable that in subjects with cochlear implants, there is an auditory sensation in the absence of physiological stimulation of the cochlear sense organ. Perhaps it is the concept of sensation that should be re-examined in the light of modern bionics. 

However, we agree that tinnitus (Jastreboff 1,2, [4]), to be such, must be perceived in the absence of any vibratory or mechanical activity at the level of the cochlea and is therefore not related to external auditory stimulation.

This concept allows us to distinguish tinnitus proper from somatosounds (subjective tinnitus) or body sounds, such as tubal sounds (movements of the tuba), often objective, which can be recorded with sensitive microphones and, unlike tinnitus, are not subjective. In somatosounds, vibratory stimulation (such as tubal stimulation, for example) is really present. Furthermore, somatosounds are not an expression of “activity within the central nervous system”. Therefore, in our opinion, it is a mistake to include subjects with somatosound in the statistics on the prevalence and/or incidence of tinnitus or of the therapeutic results of a drug or a rehabilitation method similar to that for actual tinnitus. Somatosounds, mechanical noises inside the organism produced by the vascular system, the heart, joint friction, rhythmic and involuntary muscular contractions, can also have some medical importance. It may be appropriate to operate a further subdivision that should include the pulsating noises produced, for example, by heart valve prostheses. These sounds perceived by the patient cannot be considered somatosounds in the strict sense of the term, as they are produced by an internal vibration and are not consequent on phenomena of dys-neuroplasticity. Fortunately, although the sound of heart valve prostheses is perceived by 83% of patients who carry them, only 17% showed high levels of discomfort.

To return to the topic of tinnitus proper, this, as already mentioned, involves the central auditory pathways. If the pathology of tinnitus is to be identified here, it seems legitimate to wonder whether it is not possible to define tinnitus with terms similar to those used for other diseases of the Central Nervous System. Ultimately, why treat tinnitus as a “special pathology” and not consider the hypothesis of setting it alongside other analogous neurological pathological processes? Moreover, this hypothesis would open the way for new therapeutic–rehabilitative approaches.

Examining the pathologies of the nervous system, we come across the term “dysperception”, defined in the “Treccani Dictionary of Medicine” as an “alteration of the faculty of perceiving, that is, of acquiring, through the senses, information about oneself and the surrounding world”.

Dysgraphia, dyslexia and misspelling are dysperceptive acts.

**Figure 1:**
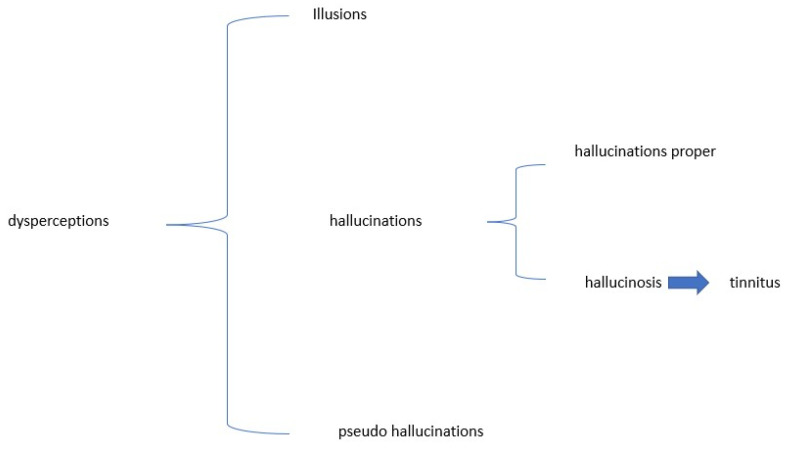
Classification of dysperception.

In the early 1800s, the French psychiatrist Jean-Étienne Dominique Esquirol classified the world of dysperceptions, distinguishing between illusions and hallucinations [5] (Shown in Figure 1). 

According to this classification, illusions are to be seen as an erroneous interpretation of a sensory stimulus, which, however, really exists (e.g., an optical illusion). If tinnitus is the perception of a sound in the absence of sound stimulation, and therefore, not a real sound, it cannot be defined as an auditory illusion.

Conversely, hallucinations take the form of perception of a non-existent stimulus, and this is the case with tinnitus.

If tinnitus falls within sensory hallucinations, it must immediately be stated that this only initially has two subdivisions. Esquirol [5] himself proposes that we differentiate hallucinations proper from hallucinosis. As regards the former, the subject perceiving the hallucinatory stimulus does not evaluate it as non-existent and behaves as if it were real, taking on consequent behaviors. For example, if he or she reports a hallucinatory vision of a ghost, he or she may engage in flight behavior.

The case of hallucinosis is different. Here, the perception of an unreal object is observed, as in hallucinations, but the person is aware of the non-reality of his or her own perception. Consequently, in this case, we will not observe, as in hallucination, a conforming behavior, but the subject will adopt a critical behavior in relation to the erroneous perception. This is what happens in the case of subjects with tinnitus, who obviously show a critical attitude towards their perception, so much so that they go to the doctor. Tinnitus could therefore be classified as hallucinosis due to perception disturbance.

Can hallucinosis be defined as pseudo-hallucination?

In “the Italian Treatise on Psychiatry” [6], perception disorders include not only hallucinosis but also pseudo-hallucination, illusion and finally hallucination.

At this point, in order to understand the difference, in addition to the already known concept of perception, we must examine that of representation and evaluate in the case in question the presence or absence of verbal expressions and/or delirious behaviors.

Representation, unlike perception, is an activity of thought that allows us to feel emotions, passions, fantasies, etc., as if they were internal objects. Therefore, perception seems to be part of the outer space. Representation, on the other hand, concerns our inner world, it is subjective, imaginary and, above all, voluntary.

Pseudo-hallucination is the result of a voluntary and imaginary mental representation whose contents do not claim to be reality. 

Tinnitus is not voluntary and is an expression not of representation but of mental perception, and therefore cannot be included among pseudo-hallucinations.

Let us now turn to the second distinctive point: delusional behavior, which is only present in hallucinations proper.

The delusional character means that, on the one hand, the dysperceptive act determines the certainty—and this does not change even when we are faced with the facts—of its actual reality, and on the other hand, that those affected present consequential behaviors, such as flight [6].

It is important to note that in the initial stages of their pathology, even tinnitus sufferers do not recognize the tinnitus stimulus as unreal and often have pseudo-delirious behaviors, such as trying to turn off the refrigerator. However, faced with the facts, they immediately take a critical attitude towards their own hallucinosis, interrupt any attitude inconsistent with reality, and indeed turn to a specialist.

Pseudo-hallucinations and hallucinations are delusional, but hallucinosis and illusions are not.

Hallucinosis mostly involves the visual and auditory senses, while hallucination can involve any sense organ. Examples of hallucinosis can be seen in Lhermitte’s hallucinosis, called peduncular, as it is located in the cerebral peduncles and gives rise to the impression of seeing colored images, such as flowers or animals, which are motionless and more frequent in the evening. It was first reported in a 72-year-old woman by Jean Lhermitte in 1992 [7].

A further distinction between hallucination and hallucinosis could be made on the basis of the more or less structured content of a person’s dysperception. It is known that structuring is high in hallucination, whereas it is reduced in hallucinosis [8]. Yet, frequently tinnitus sufferers report the perception of well-structured sounds, sometimes even endowed with a coherent and elegant musical tonality.

This topic is described by Oliver Sacks in the volume *Musicophilia* [9]. The author refers to a female patient with musical perceptions. He says her hallucinations were not psychotic but neurological, the so-called release hallucinations [that we could define as deafferentation hallucinations, like the phantom limb]. Given Mrs. C.’s deafness, the auditory part of her brain, deprived of the usual afferents, began to generate spontaneous activity on its own, which took the form of musical hallucinations, mainly musical memories of her youth. The brain needs to remain constantly active and if it does not receive its usual auditory or visual stimulation, it creates it in the form of hallucinations. Therefore, we see that Sacks, while excluding the psychotic–psychiatric nature of the observed symptoms, used the term hallucination. 

To understand the correct use of the terminology, let us turn once again to “the *Italian Treatise of Psychiatry*” [6]. The authors dwell at length on the distinction between hallucinosis and hallucinations, dedicating an entire section to this topic.

Both hallucination and hallucinosis have the characteristics of objectless perception, of the physical experience of perception, but, unlike hallucination, hallucinosis has a reduced structure, mainly affects the senses of sight and hearing, has questionable subjective certainty, is recognized and criticized by the subject, is never self-referring but neutral, recognizes an organic cause and finally does not determine a delusional attitude [6].

Should we include in the definition of tinnitus if the sound has a structured organization, unorganized acoustic perception?

Likewise, no tinnitus patient, for instance, reports hearing a pure tone at 250 Hertz, or a white noise or a narrow band noise but recognizes the perception as more or less structured, referring it to concrete examples: a bee, refrigerator noise, the wind and so on. Even the tinnitus sufferer structures and gives good shape to his or her perception. We are back in the field of Gestalt [4].

For this psychological current, each of us, in order to understand the surrounding world, tends to identify forms according to patterns that seem to be chosen by imitation, learning and sharing. Through these processes, perception and thought are organized, usually unconsciously. With particular reference to visual perceptions, among the main rules of the organization of perceived data, we recognize a good form, and this is how the perceived structure is always the simplest [10].

**Figure 2:**
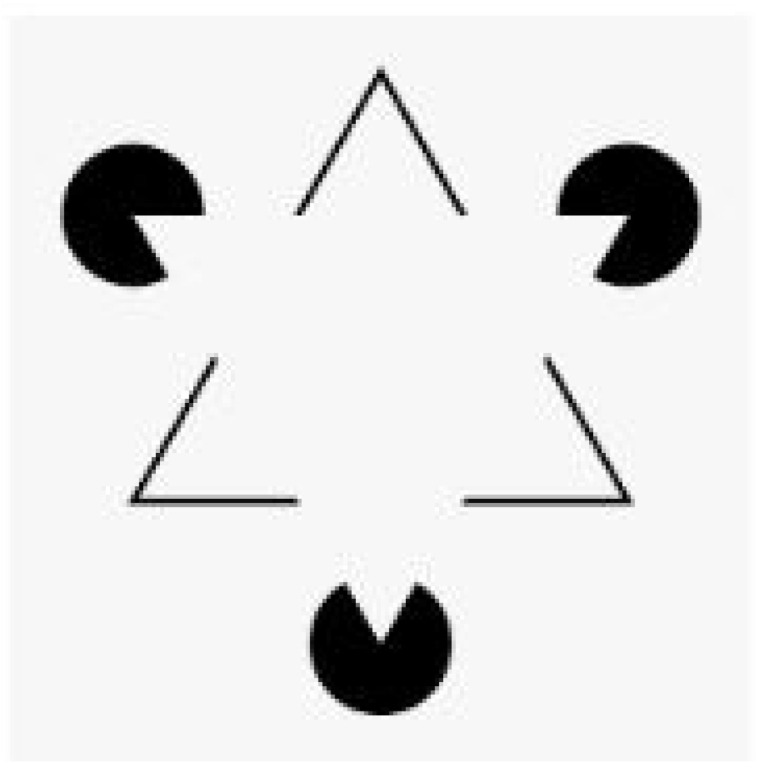
Triangle Kanizsa.

An example of a good perceptual form is the triangle of Cajetan Kanizsa [11]. In the Figure 2 (below), “we see” two white equilateral triangles, one superimposed on the other, although neither of the two triangles is actually drawn.

Ours is a brain that, on the one hand, has high energy consumption and, on the other, is structured with a social purpose. The high energy consumption will make it necessary for us both to develop the attentional function and to assemble the different pieces of information together, so as to achieve the perception a “good shape” [12]). This is one of the basic principles for understanding the aforementioned Gestalt theory. According to this school, the perception of an object is the result of the organization of the nervous system and not just of information that has reached the cochlea or the retina.

In agreement with C. Rovelli [13], we must admit that “‘things’” like ‘concepts’ are fixed points in neuronal dynamics, induced by recurrent structures in sensory inputs and in subsequent processing. Therefore, we group in a single image the set of processes that make up those organisms that other human beings are because our life is social.

We group together different pieces of information so as to provide our mind with a unique, holistic and, above all, credible concept. We represent the world by grouping its parts and breaking it up into processes that we try to make interact with each other. We need to give a “good shape”—credibility—to our observation, transforming the sensations into a coherent perception. Hence, clouds, mountains or faces themselves normally take on familiar shapes for us. For those who suffer from tinnitus, even an unstructured, meaningless sound takes on its own connotation so as to seem rich in information. This mechanism is a bit like the “Trojan Horse” that tinnitus uses in order not to be filtered, as useless information, by the filter systems of the Reticular System.

Each piece of information will therefore be first transduced by the sense organ and then processed by the entire central nervous system, so as to determine the perception of a “good shape” (the perceived structure is always the simplest), similarity (tendency to group similar elements), good continuity (all elements are perceived as belonging to a coherent and continuous whole), a background figure (all parts of a zone can be interpreted both as objects and as a background) and finally pregnancy or good shape [14]. The latter point means that if we are facing stimuli with different interpretations, such as in our case tinnitus consisting of a “meaningless” sound with a sound similar to music, we will perceive it in the simplest and most credible. Not, for example, as a frequency “fluctuating” between 1000 and 8000 Hz but more simply as a musical sound. We will not perceive a 225 Hertz frequency in narrow-band noise but the “tank” of the hydraulic system, not 4000 Hz in white noise but the noise of a mosquito, and so on. Perceptual “simplicity” will refer to Experience. These phenomena involve the amygdala, a set of nuclei located within the temporal lobe of the brain, which is decisive in the storage of emotional memory and, for this reason, according to many, sometimes responsible for the perception of tinnitus [15,16].

If we have accepted the hypothesis that tinnitus is an auditory dysperception that can be classified as hallucinosis, we must now ask ourselves what can cause dysperception.

In the introduction, reference was made to the fact that tinnitus is believed to be the result of damage to the internal hair cells of the organ of Corti of the inner ear, which is the receptor of the Peripheral Hearing System. However, this damage is sometimes so fragmented that it cannot always be documented with current audiological methods. Deafness is the consequence of damage, of a pathogenic noxa, affecting the inner ear. Almost all authors [17,18] agree that neuroplastic involvement of the central nervous system, which causes tinnitus, is in any case secondary to a peripheral auditory lesion which is more or less evident (with routine audiological systems).

M. B. Calford and R. Tweedale [18] showed that in primates, the response begins a few minutes after an experimental pharmacological amputation. In the second phase of the neuro-phlogistic process, elements of the macroglia, in particular the astrocytes, intervene. These cells are the protagonists of cortical metabolic activity since, in addition to responding to the stimulus of exogenous pro-inflammatory molecules or those produced by the activated microglia itself, they also produce growth factors and neurotrophines essential for the survival of neuronal cells. This moment represents the link between neuro-phlogosis and neuro-plasticity. The latter property [19] allows the central nervous system to change its structure in response not only to inflammatory events such as those in question but also to stimuli received from the external environment or to the normal development process of an individual. Some factors (for example, sleep and melatonin, physical exercise, trauma and cortical ischemia, steroids and lithium) stimulate neuro-plasticity. However, sometimes the neuro-phlogistic and neuro-plastic processes can be maladaptive, and the term dysneuro-plasticity is used. It should be remembered that neuro-phlogosis is very different from inflammation in other organs and systems of our organism. Restoring the nervous system is a complex process because it cannot repair itself with a scar and because the repair process must “recreate” a tissue that determines a function equivalent to that of the contralateral hemi-system in order to achieve neurological inputs that are balanced with those produced by the analogous contralateral neuron. Conversely, functional responses will be asymmetrical.

Examples of maladaptive neuro-plastic responses are also conditions of sensory dysperception, chronic pain, neuro-toxic response and, in otoneurology, tinnitus, vestibular scars and, we believe, persistent postural-perceptual dizziness (PPPD), the most common form of chronic imbalance.

The neuroplasticity of the central hearing system will mean that after hearing loss, the tonotopic mapping of the auditory cortex is altered, and a region that originally processed frequencies that are no longer perceived begins to perceive adjacent frequencies in the cortical region [20]. Neuroscience has accustomed us to the concept of “Phantom Limb” which determines the appearance of pain related to a previously amputated limb, and therefore “phantom”. Cases [19] of “Phantom Breast” have been described, down to phantom hearing, as in tinnitus. Attention is of fundamental importance for the establishment of neuroplastic phenomena [21]. When we perform tasks automatically, without paying attention to them, our brain maps change, but only for a short time. We often appreciate the ability to multitask, but dividing our attention does not result in consistent and lasting changes in our brain maps.

In the most modest cases of deafness, it is the neuro-plastic activity of the deprived cortical areas that “rebuilds” the mental image of sounds no longer perceived. However, we know that when sensory deprivation, i.e., deafness, is marked, the deprived cortical areas are no longer sufficient to compensate for the deficit, and the hippocampus is called into question which, as a depository of memory, recalls sounds, music and words already known to the subject [22].

Tinnitus is a dysperceptive hallucinosis resulting from phenomena of maladaptive neuroplasticity or dysneuroplasticity.

Hallucinosis, it has been said, is mainly visual and auditory. Visual hallucinosis is frequent in the presence of migraine auras, while auditory ones are the prerequisite for tinnitus. Having established the concept of information structuring, we will deal with “subjective certainty”, “criticality” and falsity.

Acts of Dysperception Can Take on a “Positive” or a “Negative” Character

In the 19th century, the English neurologist Hughlings Jackson [23] showed that in epilepsy there were symptoms of neurological negativity such as loss of consciousness, and positive symptoms, such as hyperactivity, hallucinations and delirium.

In migraine sufferers [24], we observe positive and negative visual disturbances (sparkling scotoma, negative scotoma, blindness, etc.).

Tinnitus can therefore be classified as a form of positive dysperception or hallucinosis of the auditory sensory system.

Negative hearing dysperceptions could be represented in otoneurology by verb–tonal dissociations, in which, with a complete auditory assessment that is within the normal limits and excluding the possible presence of a central auditory pathology, the patient reports that he or she does not correctly hear the interlocutor’s message. The maximum expression of this dysperception is non-organic hearing loss (N.O.H.L.), i.e., functional or psychogenic deafness [25]. Patients with the latter disorder, for neuropsychological reasons report an unreal hearing deficit, mostly complete unilateral deafness.

A complete definition of tinnitus must predict when it takes on pathological significance.

We have all felt a momentary ringing in one or both ears. Is it tinnitus? It certainly would be if we did not include the duration parameter in the definition of tinnitus. Tyler et al. [26] believe that tinnitus must last at least 5 min and occur more than once a week to be defined as such. Furthermore, not all cases of tinnitus require any therapeutic intervention. About 10% of the population suffers from tinnitus. Both genders are equally affected, and although tinnitus is more common in the elderly, it can occur at any age, including in childhood. However, it is only for a minority of patients that the discomfort is continuous and very significant and will require specialist support [27].

Many report having a normal coexistence with their tinnitus, which is therefore well compensated. Others find it an unbearable nuisance.

Like chronic pain [28], tinnitus is perceived as annoying or even unbearable by the patient, when our normal evaluation system of sensory stimuli (Gate keeper system) [29] is altered and attributes a dangerous character to harmless stimuli, such as a buzz. The gate-keeper system is represented in man by the complex connection between the thalamic emotional nuclei (limbic thalamus) and the nuclei of the emotional striatum, the amygdala, nucleus accumbens septi and insula, which send an incorrect evaluation to the cortical prefrontal lobes, with a consequent condition, for the tinnitus sufferer, of chronic alert [29]. Hence, the importance of describing meta-cognitive experiences in tinnitus therapy [30]. The most common tool, but not the only one, is probably the Tinnitus Handicap Inventory (THI) [31].

In these cases, some psychometric questionnaires can help us define the level of involvement of the limbic system [15,16]. 

Hence, the distinction between peripheral and central tinnitus appears rather questionable. Tinnitus originates in the periphery of the peripheral nervous system but is always organized in the structures of the central nervous system.

It is also probably superfluous to differentiate tinnitus from deafferentation (deafness) and from cross-modal involvement of the extra-lemniscal pathways. In any case, tinnitus involves initial hearing damage and therefore deafferentation and, like all perceptive acts, is in itself cross-modal and multisensory.

## 3. Results 

In agreement with one of us (Messina, 2019, proceedings of the XVIII AIOLP congress), we propose the following updated definition of tinnitus: “It is a sound not justified by any internal or external vibration, which is perceived for at least 5 min, more than once a week [32]. Tinnitus is an auditory dysperception that can be classified in the field of positive auditory hallucinosis which, as such, recognizes a pathogenesis in the phenomena of dysneuro-plasticity, resulting ‘almost always’ from an organic cochlear peripheral lesion. Being a form of hallucinosis, tinnitus can take on a coherent structure but does not determine delusional attitudes and behaviors. Tinnitus is clinically evident only if there is an altered evaluation of its signal by the fronto-limbus striatal system”.

## 4. Conclusions

Why such a complex definition of tinnitus to replace the simpler one of a “sound in the absence of external or internal vibratory stimulation”?

The ICD-10-CM classification of the Italian Ministry of Health attributes competence on tinnitus to the otolaryngologist (code H93.1); it should be realized that tinnitus, far from being a psychological problem, involves the entire peripheral and central nervous system, not excluding emotional areas.

Furthermore, if tinnitus is a process that, with a bottom-up progression, proceeds, over time, step by step, from the periphery towards cortical areas, the therapeutic approach will have on the one hand to be different depending on the level of neurological involvement and, on the other hand, to be of a neuropsychological type. It is unthinkable to assume an identical therapeutic approach for somatosounds—which are not an expression of neuroplasticity dysfunction—and for correctly defined tinnitus.

Then, only with a clear definition of tinnitus can correct epidemiological data be obtained.

Finally, we must ask ourselves whether tinnitus is hallucinosis and therefore a false learned cortical image, specifically an auditory one, and if it is possible to unlearn it with experiences that modify the altered body image, specifically auditory ones.

The use of metacognitive therapy appears to offer promising results in reducing patients’ perception of tinnitus and anxiety, reducing the significance of troubling thinking and rumination. A group appears to be a good environment for patients to share their experience and learn metacognitive techniques. Further studies are needed to test its effectiveness and replicability.

## Data Availability

Not applicable.
